# Rapid regulation of photosynthetic light harvesting in the absence of minor antenna and reaction centre complexes

**DOI:** 10.1093/jxb/eraa126

**Published:** 2020-03-09

**Authors:** Francesco Saccon, Vasco Giovagnetti, Mahendra K Shukla, Alexander V Ruban

**Affiliations:** 1 Queen Mary University of London, School of Biological and Chemical Sciences, London, UK; 2 University of Essex, UK

**Keywords:** Chlorophyll fluorescence, LHCII, NPQ, proton gradient, qE, zeaxanthin

## Abstract

Plants are subject to dramatic fluctuations in the intensity of sunlight throughout the day. When the photosynthetic machinery is exposed to high light, photons are absorbed in excess, potentially leading to oxidative damage of its delicate membrane components. A photoprotective molecular process called non-photochemical quenching (NPQ) is the fastest response carried out in the thylakoid membranes to harmlessly dissipate excess light energy. Despite having been intensely studied, the site and mechanism of this essential regulatory process are still debated. Here, we show that the main NPQ component called energy-dependent quenching (qE) is present in plants with photosynthetic membranes largely enriched in the major trimeric light-harvesting complex (LHC) II, while being deprived of all minor LHCs and most photosystem core proteins. This fast and reversible quenching depends upon thylakoid lumen acidification (ΔpH). Enhancing ΔpH amplifies the extent of the quenching and restores qE in the membranes lacking PSII subunit S protein (PsbS), whereas the carotenoid zeaxanthin modulates the kinetics and amplitude of the quenching. These findings highlight the self-regulatory properties of the photosynthetic light-harvesting membranes *in vivo*, where the ability to switch reversibly between the harvesting and dissipative states is an intrinsic property of the major LHCII.

## Teaser

We show that light harvesting control *in vivo* takes place only in the major LHCII. Allosteric-type regulation of the efficiency of this LHCII by PSII subunit S protein and zeaxanthin is also demonstrated

## Introduction

The ability to regulate the energetic fluxes in the thylakoid membranes is a crucial requisite in plants to cope with changes of environmental variables such as water and nutrient availability, cold, soil salinity, and light irradiance (Anderson *et al.*, 1988; Kramer *et al.*, 2004; Demmig-Adams *et al.*, 2012). Adaptive responses of the light-harvesting machinery take place on a relatively fast time scale and enable plants to track efficiently variations of light intensity and quality (Bonaventura and Myers, 1969; Horton *et al.*, 1996). Acting in seconds or minutes, the reversible ‘energy-dependent quenching’ component (qE) of the non-photochemical quenching (NPQ) process is the fastest adaptation to saturating light intensities and involves the formation of quenching traps to dissipate excess energy non-radiatively (Ruban *et al.*, 2012; Demmig-Adams *et al.*, 2014). By reducing the excitation energy pressure in PSII, qE minimizes the extent of photodamage from light fluctuations (Weis and Berry, 1987; Hubbart *et al.*, 2018; Murchie and Ruban, 2020) and avoids slowly reversible photoinhibitory effects associated with PSII reaction centre (RC) repair (Aro *et al.*, 1993). Understanding the molecular mechanism of qE has a great relevance for crop science research, having been shown to affect the fitness and productivity of plants (Külheim *et al.*, 2002; Kromdijk *et al.*, 2016; Głowacka *et al.*, 2018).

Numerous studies reported that the quencher in plants is formed in the trimeric light-harvesting complexes (LHC) II (encoded by the *Lhcb1–Lhcb3* genes) (Ruban *et al.*, 2012, and references therein), which represent the main fraction of pigment-binding antenna proteins serving PSII. In this context, a ‘LHCII aggregation’ model has been postulated, suggesting that qE originates from conformational changes in the LHCII that result in protein–protein interactions and consequent stabilization of the dissipative conformation (Horton *et al.*, 1991; Ruban, 2018). This model has received support from studies showing that rearrangements and clustering of antenna proteins occur *in vivo* and accompany the formation of a fast and reversible photoprotective state (Betterle *et al.*, 2009; Johnson *et al.*, 2011). The kinetics and extent of qE have been proposed to be controlled allosterically by the PSII subunit S protein (PsbS) (Li et al., 2000, 2002; Zia *et al.*, 2011; Daskalakis *et al.*, 2019) and the accumulation of the carotenoid zeaxanthin during light exposure (Demmig-Adams, 1990; Horton *et al.*, 1991). In addition to the LHCII-located mechanism, it has also been proposed that minor components of PSII, such as the monomeric LHC (CP29, CP26, and CP24, encoded by the *Lhcb4*, *Lhcb5*, and *Lhcb6* genes, respectively) and PsbS, are sites for the formation of the quencher (Li *et al.*, 2000; Niyogi *et al.*, 2005; Avenson *et al.*, 2008; Dall’Osto *et al.*, 2017; Park *et al.*, 2018). A recent study highlighted the importance of minor antenna proteins in NPQ in *Chlamydomonas reinhardtii* (Cazzaniga *et al.*, 2020). Moreover, an additional qE site has been identified within the PSII, although accounting for only a minor fraction of the process (Finazzi *et al.*, 2004; Dall’Osto *et al.*, 2017; Nicol *et al.*, 2019).

An Arabidopsis mutant that retains only LHCII, while being unable to accumulate all monomeric LHCs, has been recently reported (No Minor antennae mutant, *NoM*) (Dall’Osto et al., 2014, 2017). The maximum amplitude of NPQ in this mutant was shown to be comparable with that of wild-type (WT) plants (Dall’Osto *et al.*, 2017; Townsend *et al.*, 2018). The differences in the NPQ induction kinetics, initially assigned to the lack of monomeric LHC quenching sites (Dall’Osto *et al.*, 2017), were later revealed to be due exclusively to impaired ΔpH formation and slower zeaxanthin accumulation, which in *NoM* thylakoids significantly delay the induction of NPQ formation during the first illumination cycle (Townsend *et al.*, 2018). In this work, we aimed to characterize the kinetics, amplitude, and pH requirements of qE in plants where LHCII and ΔpH are the sole components responsible for its regulation. This was achieved by treating Arabidopsis *NoM* plants with the antibiotic lincomycin that inhibits the synthesis of chloroplast proteins, consequently causing the almost complete loss of chloroplast-encoded PSII RCs (Belgio et al., 2012, 2015; Chmeliov *et al.*, 2019). Our data show that neither the minor antenna complexes nor the RCs are required for full qE development in plants. We provide insights into the inherent ability of LHCII to control *in vivo* its light-harvesting efficiency quickly and reversibly and suggest this as the mechanism underlying qE. PsbS and the xanthophyll cycle carotenoids appear instead to be crucial allosteric modulators of the process, conferring optimal regulation of the LHCII function under physiological pH changes.

## Materials and methods

### Plant material

*Arabidopsis thaliana* Columbia (Col-0) ecotype (WT), *NoM*, and *NoM npq4* (PsbS-deficient *NoM* mutant) plants were used in this study. Seeds were sterilized in 50% ethanol and 0.1% Triton X-100, and stored for 72 h at 4 °C prior to sowing (Townsend *et al.*, 2018). Plants were grown under 180 µmol photons m^−2^ s^−1^ in a 10 h/14 h day/night photoperiod at a temperature of 22 °C. Lincomycin treatment (Belgio et al., 2012, 2015; Ware *et al.*, 2015; Sacharz *et al.*, 2017) (0.4 g l^−1^) was performed on plants at the full rosette growth stage. The treatment lasted for 2–3 weeks, until *F*_v_/*F*_m_ was ≤0.2. Treated plants were viable for several weeks, although exhibiting a significant reduction in growth rate. To induce zeaxanthin accumulation, plants were treated with white light (550 µmol photons m^−2^ s^−1^) for 2 h before measurements, and dark-adapted for 2 min before measurements, to ensure the complete re-oxidation of PSII components and relaxation of fast quenching components.

### Pigment analysis

A leaf area or ~3.15 cm^2^ was flash-frozen in liquid nitrogen, ground with a pestle, and resuspended in 0.5 ml of 80% acetone. The obtained volume was then centrifuged at 14 000 rpm for 2 min and filtered through 0.2 µm nylon filters prior to measurements. Chl *a* and *b* quantification was performed in 80% acetone as described before (Porra *et al.*, 1989). Carotenoid composition of leaf disks was evaluated through reverse-phase HPLC, using a LiChrospher 100 RP-18 column and Dionex Summit chromatography system (Ruban *et al.*, 2006).

### Isolation and characterization of protoplasts, chloroplasts, and thylakoids

Mesophyll cell protoplasts were obtained by enzymatic digestion of cell walls (Nishimura and Akazawa, 1975). After stripping the lower epidermis with adhesive tape, leaves were floated for 1 h on a solution containing 0.4 M mannitol, 20 mM KCl, 20 mM MES, 10 mM CaCl_2_, and 0.1% BSA (pH 5.5) in the presence of 1.5% cellulose Onuzuka R-10 and 0.4% macerozyme R-10 (Serva, Germany). The solution was then filtered in one layer of muslin cloth and centrifuged twice (3 min, 100 rcf, 4 °C). The obtained protoplasts were resuspended in reaction buffer containing 0.5 M sorbitol, 20 mM HEPES, 20 mM MES, 20 mM Na-citrate, 10 mM EDTA, 10 mM NaHCO_3_, 15 mM MgCl_2_, and 0.1% BSA (pH 8). Chloroplasts were obtained from osmotic shock of intact protoplasts before each measurement. Briefly, a small volume of protoplasts was diluted in ~800 µl of reaction medium not containing sorbitol. After 30 s of incubation, the solution was re-equilibrated with the 800 µl of reaction buffer containing 1 M sorbitol. Then 77 K fluorescence spectroscopy was carried out on chloroplasts isolated as previously described (Crouchman *et al.*, 2006), using 40 mM d-isoascorbate instead of 2 mM. Unstacked thylakoid membranes were prepared as previously described (Tutkus *et al.*, 2019).

### LHCII isolation and *in vitro* oligomerization

LHCIIs were isolated by flat-bed preparative isoelectric focusing of solubilized unstacked thylakoid membranes from Arabidopsis WT plants (Bassi *et al.*, 1991; Ruban *et al.*, 1994). Thylakoids corresponding to 4 mg of total chlorophyll were solubilized with 1.6% *n*-dodecyl-β-d-maltopyranoside (β-DDM) for 1 h on ice, with occasional mixing. After isolation, LHCIIs were resuspended in a buffer containing 25 mM HEPES, 0.01% β-DDM (pH 7.6). The oligomerization of the complexes was achieved through gradual removal of detergent from the bulk solution with the addition of adsorbent polystyrene beads (Biobeads, Bio-Rad, USA) (Ruban *et al.*, 2007). Chlorophyll fluorescence quenching was monitored using a PAM 101 fluorimeter, under a weak red measuring light, and the state of LHCII aggregation was evaluated recording the steady-state fluorescence emission spectrum of samples at 77 K.

### Sucrose density gradients

Seven-step exponential sucrose density gradients were prepared as previously described (Ruban *et al.*, 1999). A 20 mM HEPES, 0.06% β-DDM (pH 7.8) buffer was employed. Unstacked thylakoid membranes (total chlorophyll corresponding to 500 µg) were solubilized with 1.3% β-DDM for 1 h in the dark and on ice, using a buffer containing 0.3 M sorbitol, 1 mM EDTA, 50 mM HEPES (pH 7.6). A 1 ml aliquot was loaded into each sucrose density gradient tube and centrifuged overnight (300 000 rcf at 4 °C). Sucrose gradient bands were collected with a syringe and kept on ice until further measurements.

### SDS–PAGE

Analytical protein separation was achieved by denaturing SDS–PAGE (Laemmli, 1970; Schägger, 2006); 12% acrylamide/bis-acrylamide linear gels were employed (Mini-PROTEAN TGX Stain-Free Precast Gels, BioRad, USA). Equal volumes (20 µl) of pigmented bands collected from sucrose density gradients were loaded into each lane. Gels were stained using InstantBlue protein stain (Expedeon, USA) and scanned using a ChemiDoc Touch Imaging System (BioRad, USA).

### Fluorescence spectroscopy

Chlorophyll fluorescence quenching induction was measured using a DUAL-PAM-100 measuring system (Walz, Germany). Measurements on leaves were performed at room temperature. Protoplasts and chloroplasts were resuspended in reaction buffer in a glass cuvette (1.6 ml) at a final chlorophyll concentration of 35 µg ml^–1^ and the temperature controlled at 20 °C. Red actinic light (900 µmol photons m^−2^ s^−1^), provided by arrays of 635 nm light-emitting diodes, was applied to induce fluorescence quenching. The NPQ parameter was measured from chlorophyll fluorescence induction traces as (*F*_m_–*F*_m_')/*F*_m_' (Ruban, 2016). To block the formation of ΔpH during illumination of isolated protoplasts, nigericin (50 nM) was added before the measurements. To assess ΔpH formation in isolated chloroplasts, 9-aminoacridine (9-aa; 5 µM) was added before the start of the measurements and its fluorescence quenching was monitored (Schreiber and Klughammer, 2009). For experiments that required acidification of the reaction buffer to ensure the complete equilibration of lumen and buffer pH, nigericin (500 nM) and HCl were added simultaneously. Diaminodurene (DAD; 20–600 µM) was injected in the cuvette at the onset of actinic light to enhance ΔpH above physiological values. To investigate the effect of DAD on chloroplasts isolated from lincomycin-treated plants, 3-(3,4-dichlorophenyl)-1,1-dimethylurea (DCMU; 50 μM) was added before starting the experiment to close the residual PSII RCs and abolish the residual ΔpH formed via linear electron transport. qE dependency on 9-aa quenching was fitted using a Hill curve, defined by the equation:

$$\begin{array}{l} qE=\left(qE_{max}\times Q^{n}\right)/\left({k_{0.5}}^{n}+Q^{n}\right) \end{array}$$ (1)

where qE_max_ is the maximum theoretical value of qE, *Q* is the amplitude of 9-aa quenching, *k*_0.5_ is the half-maximal concentration constant of 9-aa quenching, and *n* is the Hill parameter.

Chl *a* fluorescence lifetime kinetics were measured to monitor the relaxation dynamics of excited chlorophylls at the *F*_m_ state (Chukhutsina *et al.*, 2019). A time-correlated single photon counting (TCSPC) set-up was employed (FluoTime 200 fluorometer, PicoQuant, Germany). Excitation was provided by a 468 nm laser diode at 20 MHz repetition rate and 0.6 mW (~30 pJ per pulse) intensity. These laser intensities are of the same order of the intensity used for PAM fluorimetry, therefore making the probability of formation of singlet–singlet annihilation artefacts negligible (Johnson and Ruban, 2009). Fluorescence was detected at 680 nm with 2 nm slit width. FluoFit software (PicoQuant, Germany) was used to analyse fluorescence lifetime data by employing a multi-exponential model with iterative re-convolution of the instrument response function (IRF; 50 ps). The χ ^2^ parameter and autocorrelation function were used to judge the quality of the fit. Average lifetimes were calculated as:

$$\begin{array}{l} \underset{i}{\sum }\;(A_{i}\times {\tau_{i}}^{2})/\underset{i}{\sum }\;(A_{i}\times \tau_{i}) \end{array}$$ (2)

where *A*_i_ is the amplitude of the ith lifetime component and τ _i_ is the respective fluorescence lifetime value (Fišerová and Kubala, 2012). At the *F*_m_ state, τ _i_ is linked to the quantum efficiency of PSII (Φ _PSII_) by the equation:

$$\begin{array}{l} \Phi_{PSII}=k_{P}/\left(1/\tau_{i}+k_{P}\right) \end{array}$$ (3)

and

$$\begin{array}{l} \tau_{i}=1/\left(k_{F}+k_{D}\right) \end{array}$$

where *k*_F_, *k*_D_, and *k*_P_ are the rate constants for fluorescence, dissipation into heat, and photochemistry of PSII, respectively. For the estimation of Φ _PSII_, *k*_P_ was assumed to be the same in leaves enriched in either violaxanthin or zeaxanthin.

Low-temperature (77 K) chlorophyll fluorescence measurements on isolated chloroplasts (10 µg ml^–1^ total chlorophyll) and LHCII were performed with a Jobin Yvon FluoroMax-3 spectrophotometer equipped with a liquid nitrogen-cooled cryostat. Excitation was defined at 435 nm with a 5 nm spectral bandwidth. The fluorescence spectral resolution was 1 nm. Spectra were normalized at their maximum value in the 675–685 nm region.

### Absorption spectroscopy

Absorption measurements were performed on an Aminco DW-2000 UV/Vis spectrophotometer (Olis Inc., USA). Absorption spectra were measured between 350 nm and 750 nm with 1 nm increments. Relative chlorophyll content (percentage relative to total amount of chlorophyll loaded) was determined by integrating the area from 600 nm to 750 nm below each spectrum. qE kinetics were obtained monitoring the changes in the absorption band at 535 nm (Δ*A*_535_; Ruban *et al.*, 1993; Townsend *et al.*, 2018), measured by the dual wavelength pair 540 nm minus 570 nm. Dual-beam mode and fixed-lambda assay collection mode were used. Leaf discs were inserted in a custom-made leaf holder and placed into a 1 cm^2^ cuvette holder positioned at 45° relative to the light beam. The temperature of the holder chamber was set to 20 °C throughout the measurements. The photomultiplier was protected by a Corning 4-96 blue-green filter and an OCL1 Cyan T400-570 mirror. A red actinic light (250 W tungsten halogen lamp, 900 μmol photons m^−2^ s^−1^) was defined by a Corning 5-58 filter.

## Results

### Making the thylakoid membranes enriched only in LHCII using lincomycin treatment

**Fig. 1.**
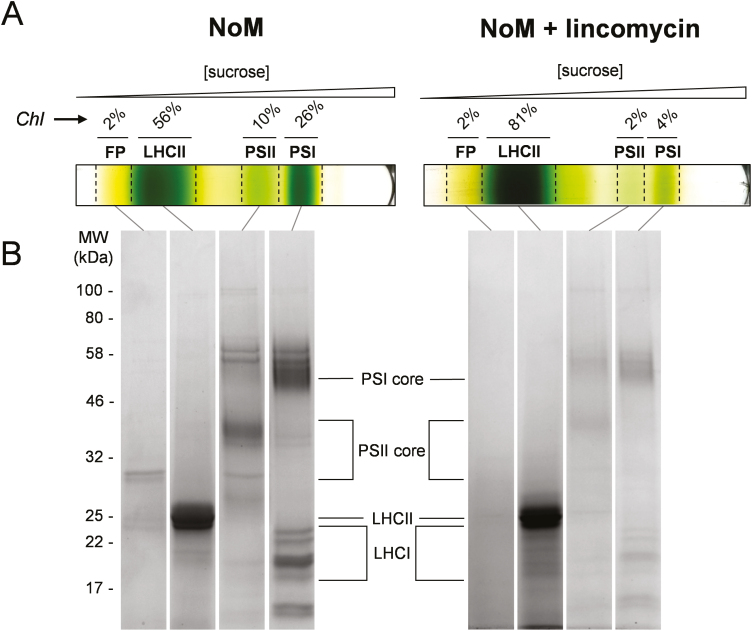
Thylakoid membrane composition of *NoM* after treatment with lincomycin. (A) Representative sucrose density gradients of solubilized thylakoid membranes isolated from *NoM* and lincomycin-treated *NoM* plants. Analysed fractions are labelled and their relative chlorophyll content is shown (the same amount of chlorophyll was loaded into each tube). (B) SDS–PAGE of the main sucrose gradient fractions. The gel lanes displayed were cropped from the same picture of the gels for clarity. Equal sample volumes were loaded into each lane. Chl, chlorophyll; FP, free pigment.

The effects of several weeks of treatment of Arabidopsis *NoM* plants with the antibiotic lincomycin, which halts translation of chloroplast-encoded proteins, are shown in Fig. 1 and compared with untreated *NoM* plants. As previously described (Belgio et al., 2012, 2015), the thylakoids of lincomycin-treated plants exhibit a strong decrease in the amount of photosystem RC proteins without negatively affecting the accumulation of nuclear-encoded antenna proteins. The separation of lincomycin-treated thylakoid membranes by sucrose density gradient ultracentrifugation revealed the presence of only one pigmented band of antenna complexes, corresponding to the major LHCII trimers (Dall’Osto *et al.*, 2014) (Fig. 1A). The lincomycin treatment resulted in a considerable reduction of the chlorophyll content in the bands associated with PSII and PSI RC components, while LHCII chlorophyll content further increased, relative to untreated *NoM* plants (Fig. 1A). Accordingly, lincomycin-treated plants revealed a severe reduction in the amount of PSII- and PSI-related polypeptides, relative to LHCII (Fig. 1B). Pigment analysis provided further support for the relative enrichment of LHCII, showing a significant decrease in the Chl *a*/*b* ratio (see Supplementary Table S1 at *JXB* online). From the ratios of chlorophyll content of the sucrose gradient bands corresponding to PSII and PSI RC of lincomycin-treated plants (Fig. 1A), we estimated a protein content reduction by ~80% and ~85% for PSII and PSI RCs, respectively. Because of the absence of PSII RC emission, 77 K fluorescence spectra from lincomycin-treated *NoM* chloroplasts present a simplified profile compared with that of WT and *NoM* chloroplasts, (Supplementary Fig. S1). Consequent to the antibiotic treatment, trimeric LHCIIs almost exclusively contribute to the fluorescence wavelengths below 710 nm. In contrast, the far-red emission band at ~728 nm is only partly due to the contribution of PSI and LHCI, whose amount is strongly reduced in these chloroplasts (Fig. 1). Moreover, a recent report of time- and temperature-resolved fluorescence emission data of chloroplasts isolated from WT lincomycin-treated plants showed that the contribution of PSI to this far-red emission is strongly abolished by the treatment (Chmeliov *et al.*, 2019). The far-red peak position appeared blue shifted by 4 nm compared with that of PSI emission from untreated WT and *NoM* plant samples (Supplementary Fig. S1C). Such a blue shift could originate either from free LHCI emitting forms (Croce *et al.*, 1998) or far-red emitting conformational states of LHCII, which have been described in single-molecule and Stark fluorescence studies (Krüger *et al.*, 2010; Wahadoszamen *et al.*, 2012). The emission band at ~700 nm, characteristic of LHCII aggregation (Mullineaux *et al.*, 1993; Miloslavina *et al.*, 2008; Chmeliov *et al.*, 2016; Akhtar *et al.*, 2019), is present in dark-adapted lincomycin-treated *NoM* samples and due to a fraction of ‘pre-aggregated’ LHCIIs (Belgio *et al.*, 2012) (Supplementary Fig. S1C), while being absent in WT and *NoM* samples (Supplementary Fig. S1A, B).

### Fast photoprotection (qE) occurs to the full extent in the absence of minor antenna complexes and reaction centres

**Fig. 2.**
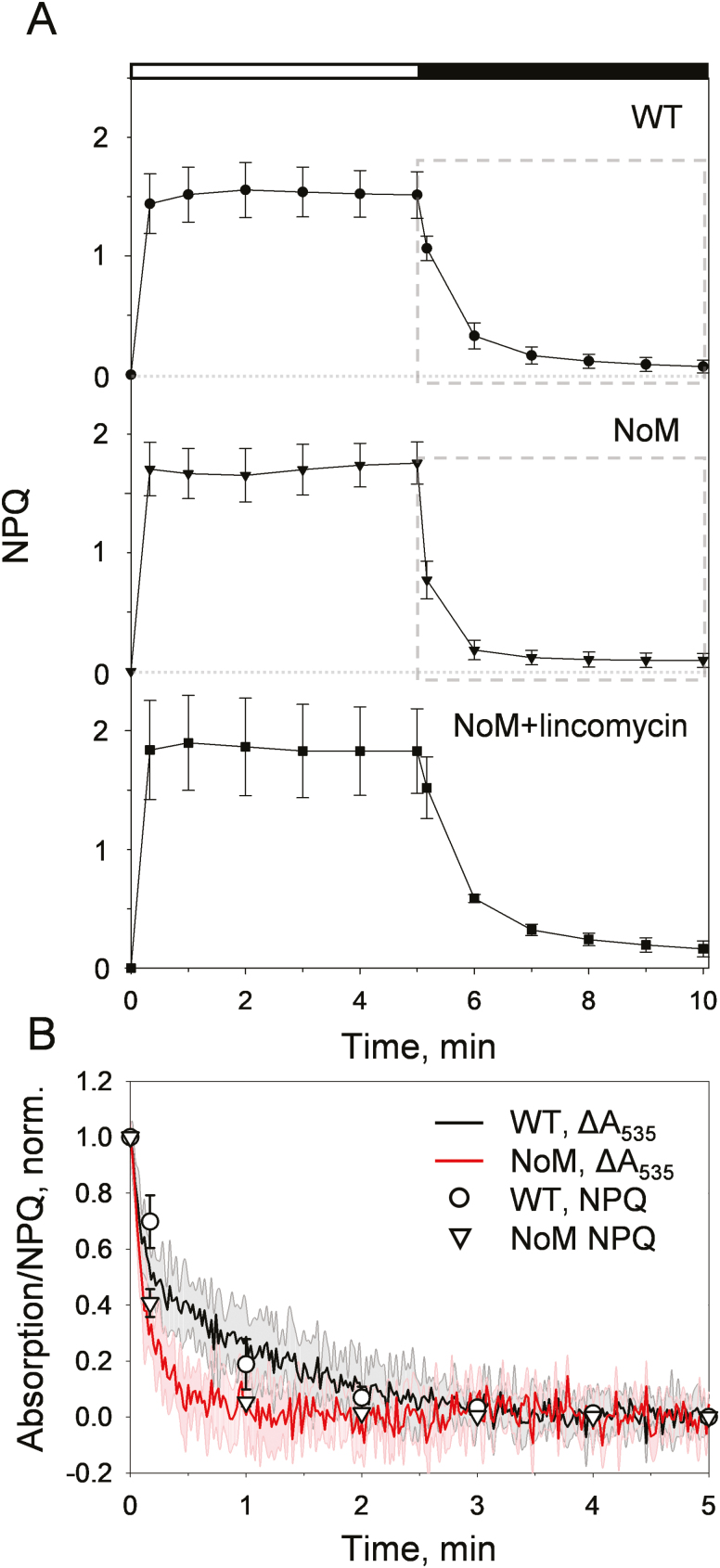
qE induction in the absence of minor LHCs and photosystem cores. (A) qE induction in WT, *NoM*, and lincomycin-treated *NoM* pre-illuminated plant leaves. Data represent averages of 3–5 replicates ±SD. Black and white bars represent periods of darkness and actinic light illumination (900 µmol m^–2^ s^–1^), respectively. Grey dashed boxes mark the values used in the comparisons shown in (B). (B) Relaxation of qE in the dark measured with NPQ values (calculated from chlorophyll fluorescence traces, symbols ±SD) and absorption changes at 535 nm (Δ*A*_535_, solid lines) for WT (black trace and open circles) and *NoM* leaves (red trace and open triangles). Solid lines represent averages of four independent absorption measurements, while shaded areas represent the SD. To compare the kinetics, NPQ values and absorption traces were offset to zero after 5 min of relaxation and normalized to the maximum value.

The ability of lincomycin-treated plants to perform NPQ was monitored through both fluorescence and absorption changes upon illumination of WT, *NoM*, and lincomycin-treated *NoM* leaves (Fig. 2A; Supplementary Fig. S2). To ensure that the maximum amplitudes of NPQ were reached, leaves were pre-illuminated to achieve violaxanthin de-epoxidation into zeaxanthin. Remarkably, the measured NPQ values were comparable for WT, *NoM*, and lincomycin-treated *NoM* plants (*P*=0.19, one-way ANOVA on NPQ values measured after 5 min of illumination; Fig. 2A). The kinetics of qE were analysed by assessing the absorption changes at 535 nm (Δ*A*_535_) that occur during the onset of quenching (Bilger and Björkman, 1990; Ruban *et al.*, 1993) (Fig 2B; Supplementary Fig. S2). Although unable to provide a direct identification of the quencher, these absorption measurements are indicative of structural changes occurring in the thylakoid membranes and have been shown to correlate with qE in WT plants (Krause, 1973). Importantly, Raman spectroscopy was used to assign the 535 nm absorption band formation to an electronic transition, rather than selective membrane scattering, which was then linked to zeaxanthin activation during light-harvesting regulation (Ruban *et al.*, 2002). A series of biochemical and theoretical works have ascribed the origin of these absorption changes to dimers of zeaxanthin forming during clustering of LHCs (Bilger and Björkman, 1990; Ruban and Horton, 1992; Billsten *et al.*, 2005; Duffy *et al.*, 2010; Ilioaia *et al.*, 2011). Here, both fluorescence and absorption measurements revealed a quick induction and reversibility of quenching, typical of qE, for WT, *NoM*, and lincomycin-treated *NoM* plants (Fig. 2A, B; Supplementary Fig. S2). Notably, in the absence of minor antennae, the kinetics of qE relaxation were accelerated compared with those of WT plants (Fig. 2B), making the qE switch quicker and possibly less wasteful, a trait sought after in recent bioengineering efforts towards crop productivity enhancement (Kromdijk *et al.*, 2016). This feature could be due to the lack of structural organization at the level of the PSII supercomplex (Dall’Osto *et al.*, 2014; Townsend *et al.*, 2018), which poses some constrains to qE in WT plants and highlights the intrinsic capability of LHCII to quickly and reversibly switch between light-harvesting and photoprotective states. This feature, however, was lost after the lincomycin treatment (Fig. 2A; Supplementary Fig. S2C). The slower relaxation of qE and Δ*A*_535_ observed in lincomycin-treated *NoM* plants relative to untreated *NoM* plants suggests that the proton gradient may be dissipated more slowly upon antibiotic treatment. The transient relaxation of quenching, previously observed in *NoM* plants within 1–3 min of illumination (Dall’Osto *et al.*, 2017; Townsend *et al.*, 2018), was also found here (Fig. 2A) and more prominently during measurements of Δ*A*_535_ kinetics from zeaxanthin-enriched leaves (Supplementary Fig. S2), while instead being almost absent in the fluorescence traces (Fig. 2A). To some extent, the linear correlation between fluorescence quenching and Δ*A*_535_ upon light exposure appeared to be lost in *NoM* and lincomycin-treated *NoM*, as already noticed in a previous report on single antisense and knockout mutants (Johnson *et al.*, 2009). Possibly, this reflects the unusual uncoupling of LHCII from the bulk antenna system observed in *NoM* leaves in response to illumination, which results in a transient loosening of intertrimer connections and thus in the observed alteration of the absorption kinetics (Dall’Osto *et al.*, 2014; Townsend *et al.*, 2018).

**Fig. 3.**
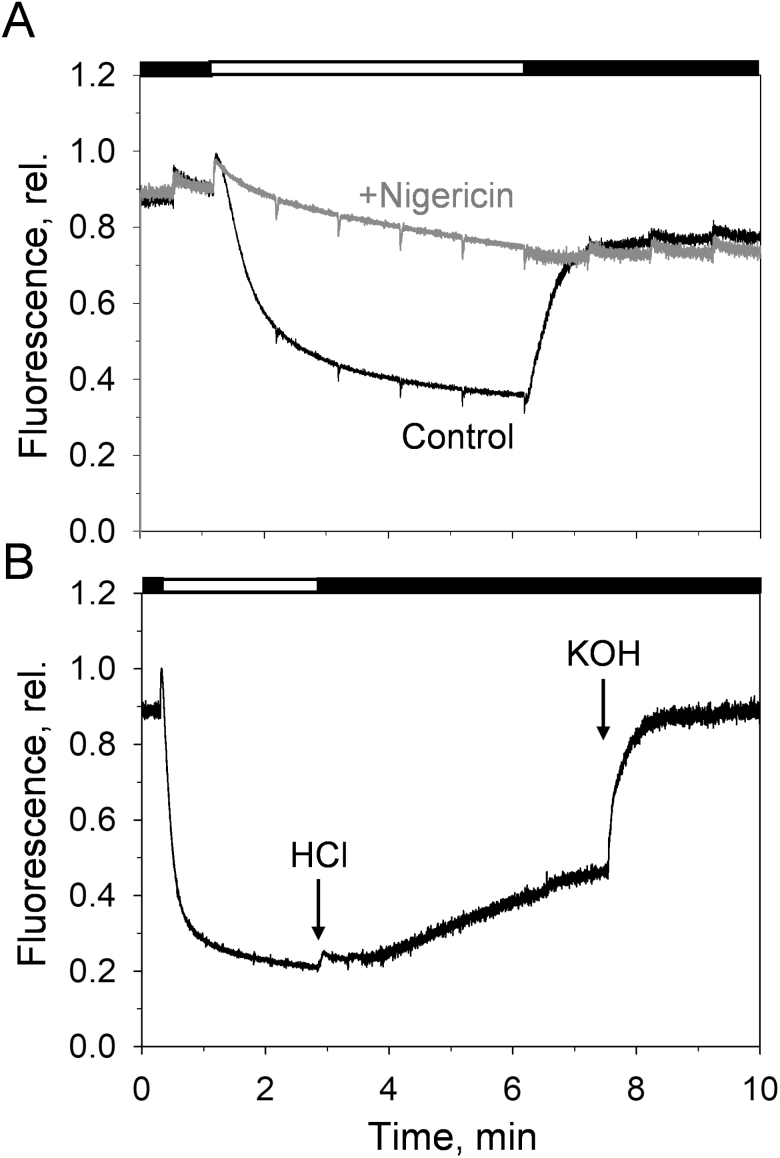
ΔpH dependence of the qE response in protoplasts and chloroplasts isolated from lincomycin-treated *NoM* plants. (A) Chlorophyll fluorescence induction traces of protoplasts in the presence (grey line) or absence of nigericin (black line, control). Traces are the average of three independent measurements, normalized at the maximum of fluorescence after the onset of actinic light. A saturating pulse was applied every minute to retrieve the NPQ values. (B) Chlorophyll fluorescence induction trace of chloroplasts exposed to bulk pH variations. After illumination, the pH was lowered to 5 by adding HCl. In the dark, the pH was restored to 8 by adding KOH. Black and white bars represent periods of darkness and actinic light illumination (900 µmol m^–2^ s^–1^), respectively. A saturating concentration of nigericin (500 nM) was added with HCl to ensure complete equilibration of lumen and bulk pH.

Overall, our data indicate that the qE formation is comparable across the plant mutants and treatments analysed. We therefore addressed whether qE in lincomycin-treated *NoM* mutants is triggered, similarly to WT plants, by the trans-thylakoid ΔpH generated by the proton gradient across the thylakoid membrane. In order to do so, we isolated protoplasts with a gentle procedure to preserve the intactness of the chloroplasts and tested the effect of the ionophore nigericin on qE formation. We found that ΔpH dissipation by nigericin inhibited qE (Fig. 3A). The relaxation of qE could be largely prevented by lowering the pH of a quenched sample despite being in the dark (pH 5, Fig. 3B), thus mimicking the lumen acidification occurring during high-light exposure. This is consistent with previous results on WT chloroplasts (Johnson and Ruban, 2011). Additionally, quenching could be reversed by increasing the pH (pH 8, Fig. 3B). These findings suggest that lincomycin-treated *NoM* mutants, with a photosynthetic apparatus mainly comprised of LHCII trimers, are able to sense pH changes that initiate qE comparably with WT plants.

### Zeaxanthin modulates the kinetics of qE and affects the *F*_m_ level in lincomycin-treated *NoM* plants

**Fig. 4.**
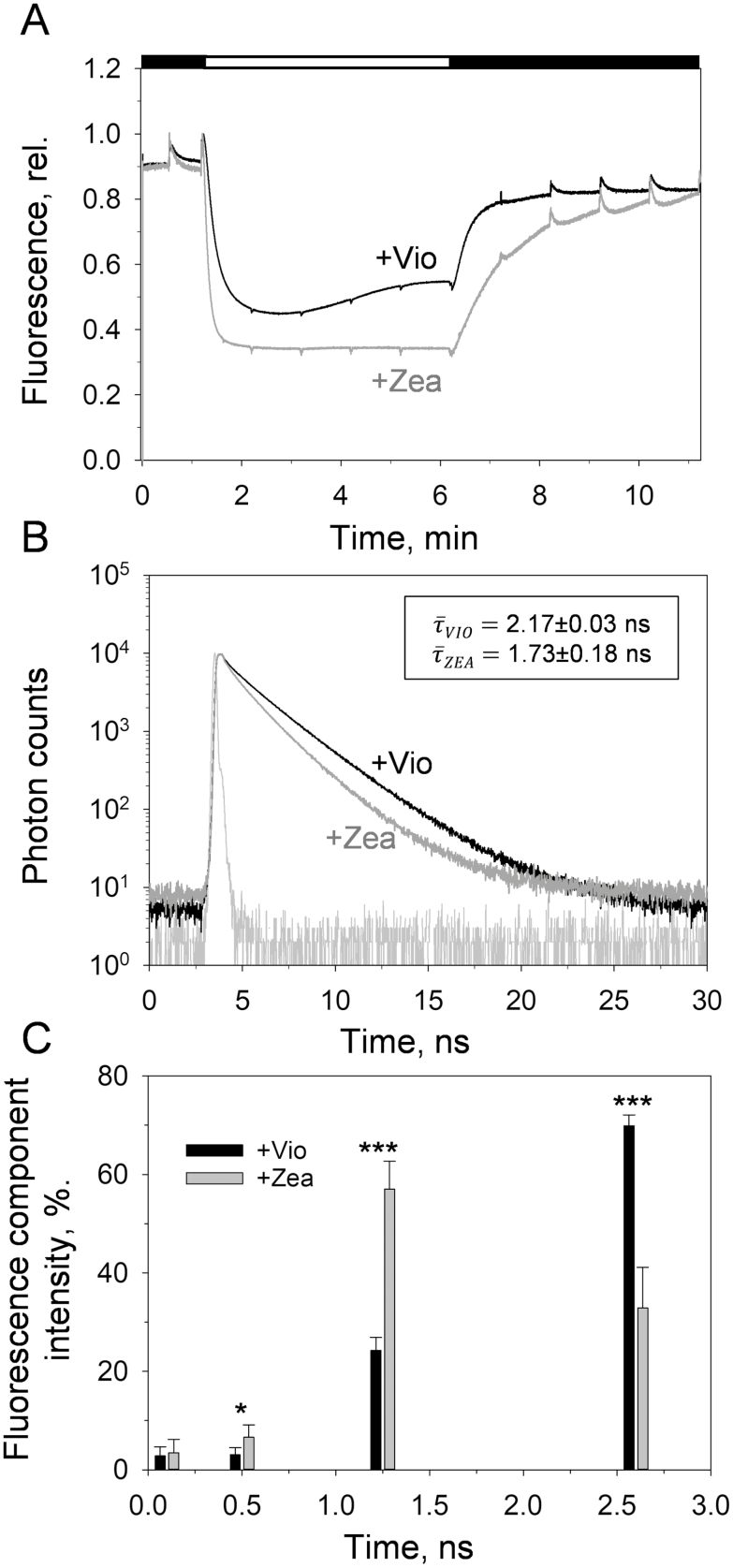
Zeaxanthin modulation of qE in leaves of lincomycin-treated *NoM* plants. (A) Fluorescence quenching induction traces of leaves from lincomycin-treated *NoM* plants, either dark adapted (+violaxanthin, black line) or pre-illuminated (+zeaxanthin, grey line). Each trace is the average of at least four independent measurements. A saturating pulse was applied every minute to retrieve the NPQ value. (B) Chl *a* fluorescence lifetime measurements of violaxanthin- (black) and zeaxanthin-enriched (grey) leaves of lincomycin-treated *NoM* plants. Prior to measurements, leaves were infiltrated with 50 µM DCMU, to fix the *F*_m_ state. Traces are averages of at least four independent measurements. The instrument response function (IRF) trace is shown in light grey. (C) Intensity-weighted components (%) obtained from chlorophyll fluorescence lifetime traces shown in (B). Lifetime components were calculated after iterative reconvolution of the IRF (see Equation 2 in the Materials and methods). The fitting procedure yielded good results using four lifetime components (0.1, 0.5, 1.25, and 2.6 ns). The fastest component originates from PSI contribution (Chukhutsina *et al.*, 2019), while the other components were attributed to LHCs in different quenching conformations (Krause and Weis, 1991; Belgio *et al.*, 2012). Error bars represent the SD and asterisks indicate statistical significance (two-tailed *t*-test: *P<0.05, ***P<0.001). Vio, violaxanthin; Zea, zeaxanthin.

To understand the effect of the selective enrichment of violaxanthin or zeaxanthin on qE in relation to the altered presence of minor LHCs and RCs, fluorescence inductions were measured on lincomycin-treated *NoM* leaves either after prolonged dark adaptation or after pre-illumination to induce zeaxanthin accumulation (Fig. 4A). We found that fluorescence quenching also occurred in the presence of violaxanthin; however, greater quenching was achieved upon zeaxanthin accumulation (NPQ=1.22±0.36 and 1.94±0.35 for violaxanthin- and zeaxanthin-enriched leaves, respectively, *P*<0.01, two-tailed *t*-test; Fig. 4A). Additionally, qE formation became faster, while relaxing more slowly, once zeaxanthin was accumulated, in line with previous observations in WT plants (see references in Ruban *et al.*, 2012).

The slow kinetics of epoxidation of zeaxanthin back to violaxanthin are known to result in sustained (i.e. slowly relaxing) quenching components (Nilkens *et al.*, 2010). Zeaxanthin synthesis was previously shown to have a long-lasting impact on the arrangement of LHCs, causing a decrease in the maximum fluorescence yield attainable in the dark (*F*_m_ state) (Johnson *et al.*, 2010). This trait was proposed to confer a ‘memory of illumination’ to plants, which in nature are often primed to promptly dissipate excess energy due to exposure to frequent light fluctuations and other abiotic stresses (Ruban and Johnson, 2010; Demmig-Adams *et al.*, 2012). We therefore investigated the occurrence of such a feature in lincomycin-treated *NoM* plants by measuring the chlorophyll fluorescence lifetime on leaves enriched in either violaxanthin or zeaxanthin, and fixed in the *F*_m_ state (Fig. 4B, C). The average fluorescence lifetime value $\left(\bar{\tau }\right)$ for zeaxanthin-enriched leaves was reduced from 2.17±0.03 ns to 1.73±0.18 ns with respect to violaxanthin-enriched leaves, matching the data obtained on WT plants (Johnson and Ruban, 2009).If we assume a value of 0.8 for quantum efficiency (ΦPSII) of dark-adapted PSII in WT plants, the experimentally determined reduction in $\bar{\tau }$ accounts for a value of Φ _PSII_ of 0.76, which is in line with values previously reported on zeaxanthin-enriched leaves (Johnson and Ruban, 2009) (see Equation 3 in the Materials and methods). Therefore, the zeaxanthin-mediated regulation of qE and the effects of its accumulation in thylakoids appear to be independent of the presence of minor antennae and RCs.

### PsbS shifts the ΔpH dependency of qE in lincomycin-treated *NoM* chloroplasts

**Fig. 5.**
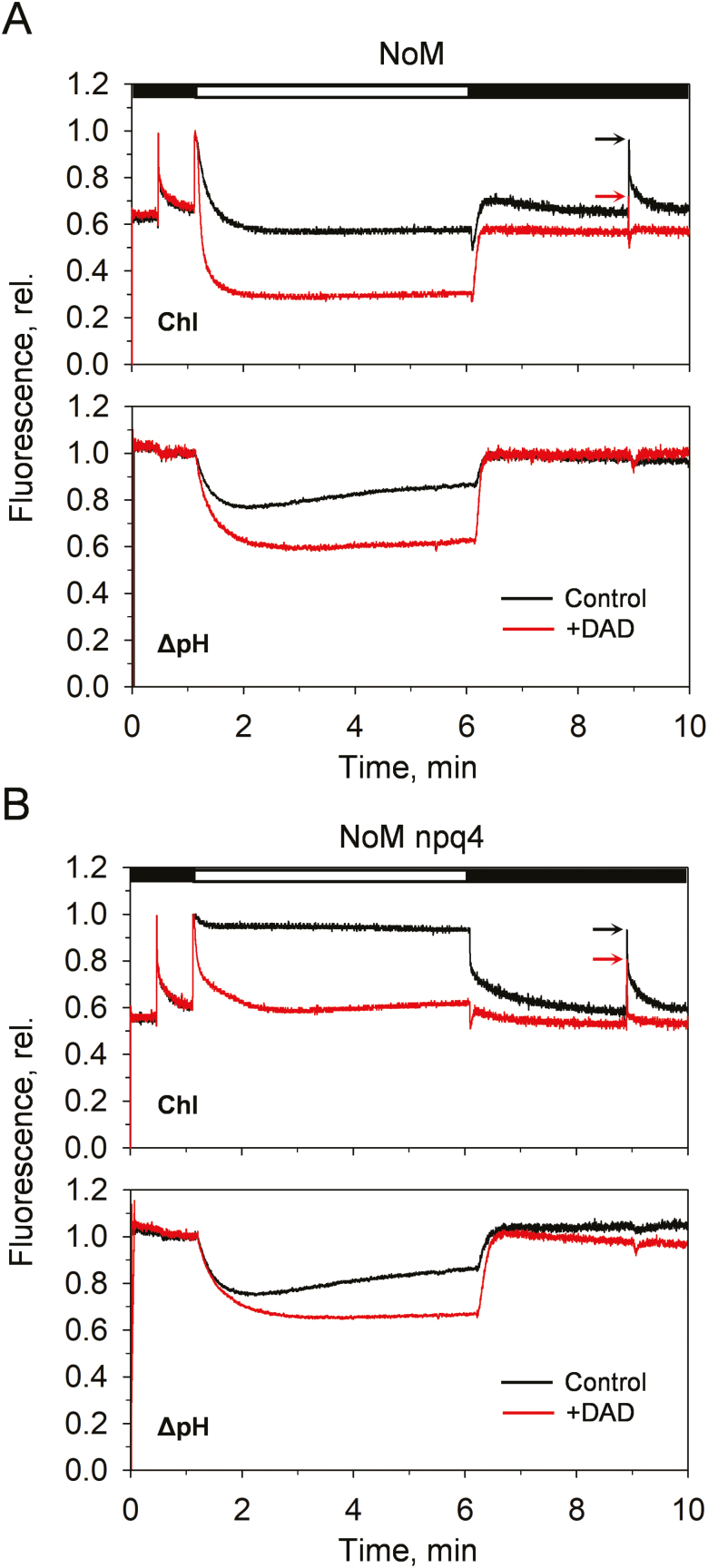
Effects of enhancing ΔpH with diaminodurene (DAD) on the amplitudes of qE in *NoM* and *NoM npq4* (devoid of PsbS; –PsbS). Representative PAM traces of chlorophyll (Chl) fluorescence and ΔpH induction from *NoM* (A) and *NoM npq4* (B) chloroplasts. ΔpH was measured by the fluorescence quenching of 9-aminoacridine (9-aa) added before each measurement (5 µM). DAD (100 µM) was added to the suspension at the beginning of the illumination period (red lines). Arrows indicate the *F*_m_ values assessed by saturating pulses after 3 min of relaxation in the dark.

PsbS is essential *in vivo* for the efficient modulation of qE (Li *et al.*, 2000). Recent biochemical and computational analyses showed that PsbS senses pH variations that activate qE (Li *et al.*, 2004; Daskalakis and Papadatos, 2017), identified the LHCII complex as its main interaction partner (Wilk *et al.*, 2013; Gerotto *et al.*, 2015; Correa-Galvis *et al.*, 2016; Sacharz *et al.*, 2017), and enabled the modelling of PsbS conformational changes in response to low pH (Wilk *et al.*, 2013; Gerotto *et al.*, 2015; Correa-Galvis *et al.*, 2016; Daskalakis and Papadatos, 2017; Sacharz *et al.*, 2017; Liguori *et al.*, 2019). Nonetheless, the mechanism of action of PsbS remains elusive. It has been proposed either to carry the quencher, possibly an activated zeaxanthin molecule (Li *et al.*, 2000; Aspinall-O’Dea *et al.*, 2002; Ruban *et al.*, 2002; Ma *et al.*, 2003; Niyogi *et al.*, 2005), or to be a modulator of qE, whose protonation brings about conformational changes and rearrangements of the antennae that form the quencher(s) (Bonente *et al.*, 2008; Kiss *et al.*, 2008; Betterle *et al.*, 2009; Johnson *et al.*, 2011). Therefore, we investigated how this crucial protein modulates qE in LHCII-enriched thylakoid membranes. Figure 5 shows the chlorophyll fluorescence induction of *NoM* chloroplasts, in the presence or absence of PsbS (*NoM* and *NoM npq4* mutants, respectively). As in WT plants, the qE is largely impaired in the absence of PsbS in *NoM* mutants (Li *et al.*, 2000) (Fig. 5B). When the ΔpH is further increased by adding the proton shuttle DAD, qE is enhanced in *NoM* (Fig. 5A) and restored in the *NoM npq4* mutant (Fig. 5B). Strikingly, these results are similar to those previously reported while assessing the role of PsbS in plants containing minor antenna complexes (Johnson and Ruban, 2011). Overall, they are in agreement with studies showing that the main functional interaction partners of PsbS are trimeric LHCII (Correa-Galvis *et al.*, 2016; Daskalakis and Papadatos, 2017; Sacharz *et al.*, 2017) and highlight the relevance of PsbS for shifting the sensitivity of LHCII quenching to higher, and physiological, values of lumen pH (Johnson and Ruban, 2011; Daskalakis and Papadatos, 2017).

**Fig. 6.**
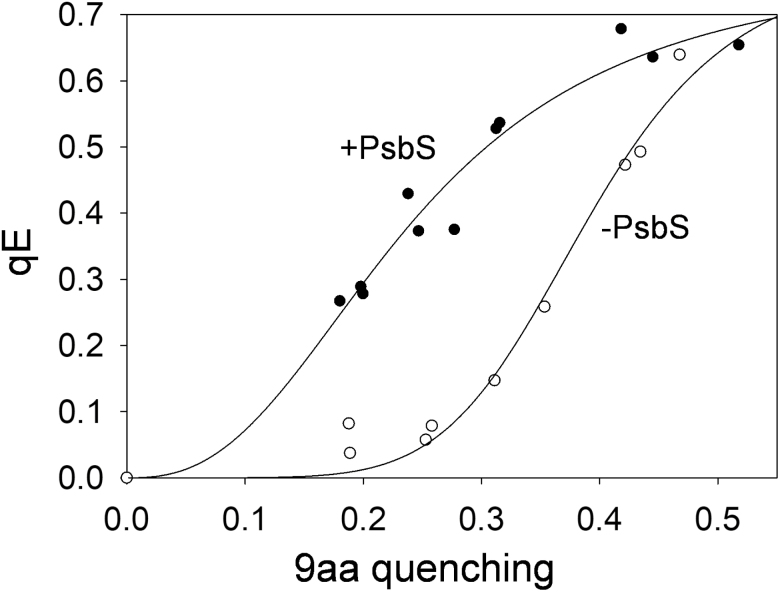
Effect of PsbS on the ΔpH dependence of qE. Relationship between reversible chlorophyll fluorescence quenching [qE, (*F*_mrelaxed_–*F*_m_')/*F*_mrelaxed_] and 9-aa fluorescence quenching in *NoM* (black dots, +PsbS) and *NoM npq4* (–PsbS) chloroplasts. The data range was obtained by changing either the actinic light intensity or the DAD concentration. Data were fitted to the Hill equation provided in the Materials and methods (*R*^2^=0.96 and 0.98 for *NoM* and *NoM npq4* chloroplasts, respectively).

Figure 6 shows the dependency of qE over a ΔpH range, obtained by varying the intensity of actinic light or the amount of DAD added to chloroplast samples. The absence of PsbS resulted in higher p*K* values of qE (e.g. the 9-aa quenching value at half qE maximum corresponded to *k*_0.5_=0.24±0.03 and 0.39±0.06 for *NoM* and *NoM npq4*, respectively) and changed the cooperativity of the process (Hill parameter *n*=2.58±0.69 and 6.17±0.62 for *NoM* and *NoM npq4*, respectively; Fig. 6). This result indicates the same cooperative nature of qE quenching in *NoM* and WT plants (Johnson and Ruban, 2011).

**Fig. 7.**
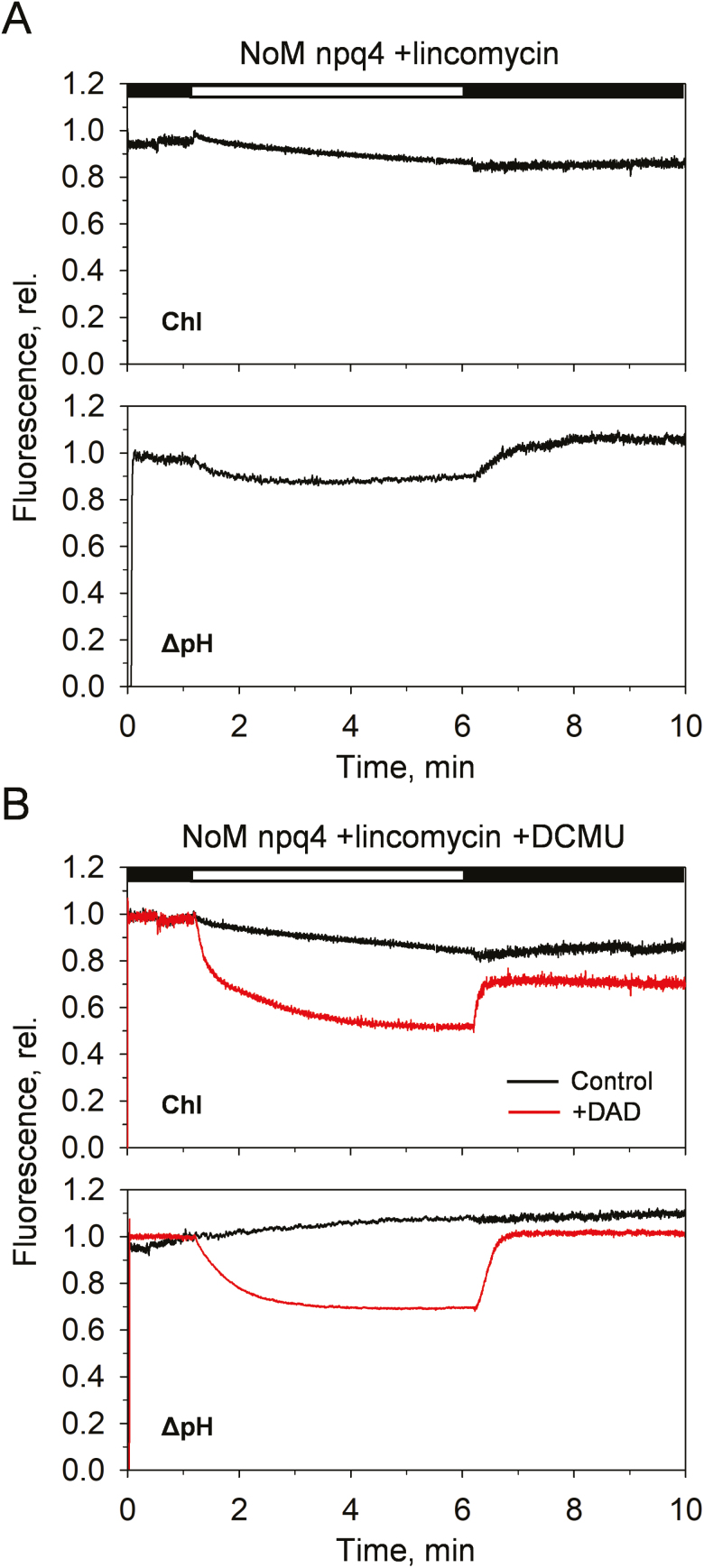
Effects of enhancing ΔpH with diaminodurene (DAD) on the amplitudes of qE of lincomycin-treated *NoM npq4* in the presence of DCMU. (A) Representative PAM traces of chlorophyll (Chl) fluorescence and ΔpH induction from *NoM npq4* chloroplasts. ΔpH was measured by the fluorescence quenching of 9-aminoacridine (9-aa) added before each measurement (5 µM). (B) The effect of DAD (red traces) and 3-(3,4-dichlorophenyl)-1,1-dimethylurea (DCMU) on chloroplasts of lincomycin-treated *NoM npq4* plants. DCMU (50 μM) was added prior to experiments to close the remaining fraction of PSII reaction centres. DAD (100 µM) was added at the beginning of the illumination phase.

The same phenotype described for *NoM* plants devoid of PsbS (Fig. 5B) was also observed in lincomycin-treated *NoM npq4* plants (Fig. 7). This suggests that PSII supercomplex organization, as well as the presence of minor antennae and RCs, is not required for PsbS activity. In this context, DCMU was used to abolish the contribution of PSII RCs in ΔpH generation (Fig. 7B). Despite the fact that PSII RCs were blocked, qE was restored by ΔpH enhancement (i.e. DAD addition).

## Discussion

In this study, we dissected the contribution of all the components suggested to take part in the qE mechanism. We applied an increasingly reductionist approach that enabled us to describe the ability of thylakoid membranes devoid of RCs, minor antennae, and PsbS to undergo qE changes, provided a sufficiently large ΔpH is induced (Fig. 7B).

Several models have been hypothesized to explain qE molecular mechanisms in plants (Holzwarth *et al.*, 2009; Ruban, 2016; Dall’Osto *et al.*, 2017; Bennett *et al.*, 2019; Nicol *et al.*, 2019). While both major and minor antenna complexes have been proposed as sites of qE formation (Holzwarth *et al.*, 2009; Ruban, 2016; Dall’Osto *et al.*, 2017; Park *et al.*, 2018), it is clear that a conspicuous fraction of qE occurs even when minor antennae are not present (Dall’Osto *et al.*, 2017; Townsend *et al.*, 2018). When zeaxanthin is accumulated, the residual differences disappear (Dall’Osto *et al.*, 2017; Townsend *et al.*, 2018). The current work provides further evidence for the lack of contribution of monomeric antennae to qE, showing that both qE induction and Δ*A*_535_ occur at similar amplitudes and even with faster kinetics of recovery in *NoM* relative to the WT (Fig. 2). Importantly, by altering the trans-thylakoid ΔpH, we revealed for the first time the presence of a cooperative proton sensitivity of the qE mechanism in the exclusive presence of the major LHCII (see Figs 5–7), similarly to what is already documented in WT plants (Johnson and Ruban, 2011). Despite playing no role in qE, the importance of minor antenna complexes for the regulation of PSII energy balance cannot be neglected. It was indeed shown that minor antennae are essential for supercomplex assembly, thylakoid reorganization, membrane stacking, and excitation energy transfer, and likely to be involved in electron/proton transport (Kovács *et al.*, 2006; de Bianchi et al., 2008, 2011; Van Oort *et al.*, 2010; Dall’Osto et al., 2017, 2020; Townsend *et al.*, 2018; Cazzaniga *et al.*, 2020). Altogether, these data underline the key role of minor LHCs in the efficient and robust design of the plant photosynthetic machinery. A recent report by Nicol *et al.* (2019) showed that the absence of the Lhcb1 and Lhcb2 polypeptides brings about a 60% reduction of the NPQ capacity in leaves, in line with a substantial contribution of LHCII to the process. It was proposed that the residual fraction of PsbS-dependent qE originates from PSII RC quenching. By investigating PSII RC-devoid (lincomycin-treated) plants (e.g. Fig. 2) and using DCMU to inhibit PSII RC activity (Fig. 7B), here instead we expose the complete independence of qE from the presence of PSII RCs.

The activity of zeaxanthin is entirely linked to the major LHCII trimers (Fig. 4).Although it still cannot be excluded that zeaxanthin may play a direct role in quenching of excess excitation energy (Park *et al.*, 2018), this and other studies suggest a rather indirect role for zeaxanthin (Johnson *et al.*, 2009; Xu *et al.*, 2015; Tutkus *et al.*, 2019) and frame it in the context of allosteric regulation of the LHCII conformational switch (Horton *et al.*, 1991; Ruban, 2018). We show indeed that zeaxanthin enhances the amplitudes and affects the dynamics of qE, without however precluding LHCII-enriched plants undergoing reversible quenching if absent (see Fig. 4). The ability of PsbS to modulate qE was also shown to be conserved even in the absence of RCs and minor LHCs (see Figs 5–7). The dependency found between qE and ΔpH shows that the protein is not essential for qE, but is pivotal in tuning its sensitivity to protons and increasing the p*K* value of LHCII (Johnson and Ruban, 2011; Liguori *et al.*, 2019).

ΔpH in the thylakoids is the intermediary between the harvesting of sunlight energy and its storage in a stable chemical form (ATP). High values of ΔpH can, however, be limiting, causing the inhibition of electron transport (Schönknecht *et al.*, 1995), or can even be detrimental, being linked to photoinhibitory processes (Spetea *et al.*, 1997). It is likely, therefore, that ΔpH is regulated within narrow limits (Schönknecht *et al.*, 1995; Kramer *et al.*, 1999). In this context, allostery and cooperativity in the control of qE have profound physiological implications. LHCII trimers alone have a limited ability to sense protons (Petrou *et al.*, 2014) and are in fact irresponsive to physiological ΔpH variations. PsbS and zeaxanthin act synergically as allosteric modulators of the proton sensitivity of LHCII, tuning its activation to less acidic luminal pH values. Thus, both factors add flexibility to the qE mechanism and make it tunable in relation to environmental conditions and metabolic needs. The cooperativity between LHCII units offers an additional control to the qE switch, allowing its complete induction within small pH changes. Superimposed on this allosteric regulation of light harvesting, natural genetic variation of PsbS and zeaxanthin-related genes give different plant species a high adaptability to diverse natural environments (Demmig-Adams *et al.*, 2006; Rungrat *et al.*, 2019).

### Conclusions

Our results unveil the inherent *in vivo* ability of the major LHCII antenna trimers to self-tune their light-harvesting function in the thylakoid membrane, in the absence of minor antennae and RC complexes. These results narrow down the essential requirements for qE to the major trimeric LHCII (site) and ΔpH (trigger). Here the allosteric model of control of the light-harvesting function gains further support as the mechanism of qE (Horton *et al.*, 1991; Ruban, 2018). Plants must regulate their photosynthetic machinery in a tight range of physiological ΔpH values to guarantee the occurrence of photochemistry and simultaneously avoid photoinhibition. Consequently, the process to control the light-harvesting efficiency of LHCII needs to be itself modulated, making it highly responsive in a narrow pH range. For this purpose, allostery and cooperativity, that are pervasive features in biological systems (Whitty, 2008), underline the regulation of qE in plants. Offering a dynamic adaptation of qE to the light environment, the allosteric control defined by PsbS and zeaxanthin is necessary to preserve its ‘economic’ nature (Belgio *et al.*, 2014; van Amerongen and Chmeliov, 2020). The function of LHCII is therefore optimally modulated to ensure the correct balance between protection and photosynthetic productivity.

## Supplementary data

Supplementary data are available at *JXB* online.

Table S1. Pigment composition of leaves from WT, *NoM*, and lincomycin-treated *NoM* (+ or – zeaxanthin).

Fig. S1. 77 K fluorescence spectra of dark-adapted chloroplasts isolated from WT, *NoM*, and lincomycin-treated *NoM* plants.

Fig. S2. 535 nm absorption changes (Δ*A*_535_) associated with qE in leaves of WT, *NoM*, and lincomycin-treated *NoM*.

eraa126_suppl_Supplementary_Figure_Table
