# Supplementary material for: Rapid regulation of photosynthetic light harvesting in the absence of minor antenna and reaction centre complexes
Source: J Exp Bot. 2020 Mar 9;71(12):3626–37. doi: 10.1093/jxb/eraa126 (PMC7307847; doi:10.1093/jxb/eraa126)
Supplement: eraa126_suppl_Supplementary_Figure_Table [file eraa126_suppl_supplementary_figure_table.pdf]

Rapid regulation of the photosynthetic light harvesting in the absence of minor antenna complexes and reaction centres

Francesco Saccon, Vasco Giovagnetti, Mahendra K. Shukla and Alexander V. Ruban*

Queen Mary University of London, School of Biological and Chemical Sciences, London, UK

**corresponding author: a.ruban@qmul.ac.uk*

Supplementary materials

Supplementary table 1. Saccon et al.

	Chl a/b	%N/Car	%V/Car	%A/Car	%L/Car	%Z/Car	%β-car/Car	DEP (%)
WT	3.45 ± 0.09	12.47 ± 0.42	10.09 ± 2.46	-	64.78 ± 1.24	-	22.48 ± 0.9	-
NoM	3.02 ± 0.34	14.3 ± 0.96	8.27 ± 0.26	-	67.73 ± 1.35	-	18.47 ± 0.93	-
NoM+lincomycin (v)	2.08 ± 0.07	16.04 ± 0.29	11.15 ± 0.39	0.629 ± 0.57	54.95 ± 1.69	-	7.4 ± 1.42	2.56 ± 2.3
NoM+lincomycin (z)	2.05 ± 0.07	15.88 ± 0.32	3.83 ± 0.94	3.63 ± 0.66	59.96 ± 0.83	2.57 ± 0.447	6.35 ± 0.78	43.73 ± 6.96

Supplementary Table 1: Pigment composition of *Arabidopsis* leaves. Chlorophyll and carotenoid content in leaves of WT, *NoM* and lincomycin-treated *NoM* plants, the latter either enriched in violaxanthin (v) or zeaxanthin (z). Chl a/b, chlorophyll *a*/*b* ratio; %N(V,A,L,Z,β-car)/Car, percentage of neoxanthin (violaxanthin, antheraxanthin, lutein, zeaxanthin, β-carotene) relative to total carotenoid content; DEP (%), de-epoxidation parameter calculated as ([zeaxanthin]+0.5*[antheraxanthin])*100/([zeaxanthin]+0.5*[antheraxanthin]+ [violaxanthin]). Data are the average of 4 independent experiments ± SD.

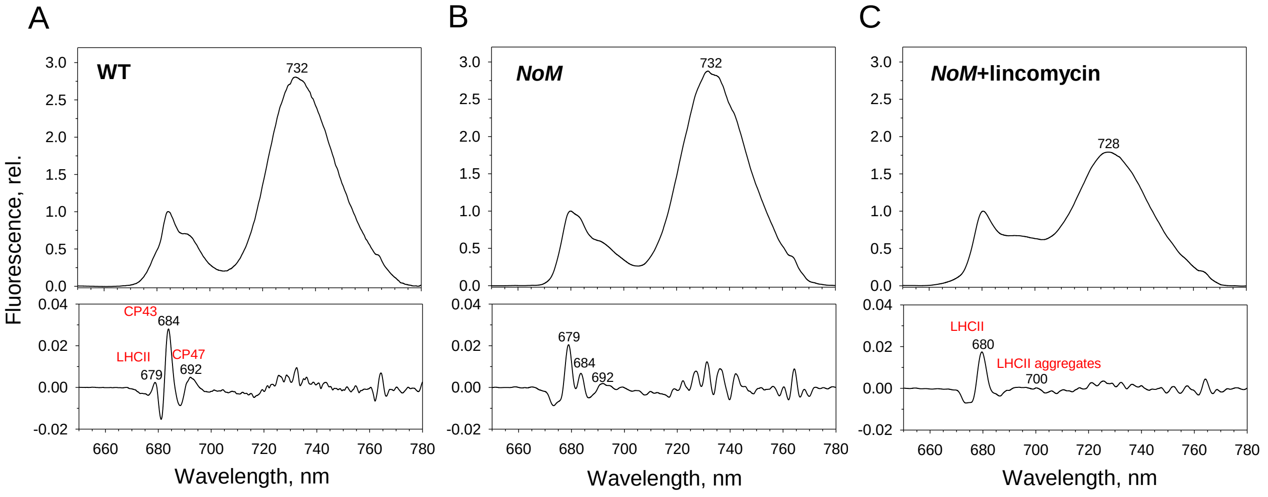

Supplementary Figure 1: 77K fluorescence emission spectra of chloroplasts isolated from dark-adapted WT, *NoM* and lincomycin-treated *NoM* plants. The corresponding second derivative peak analysis is shown below each spectrum. Excitation wavelength was set to 436 nm. Data are averages of 3 replicates.

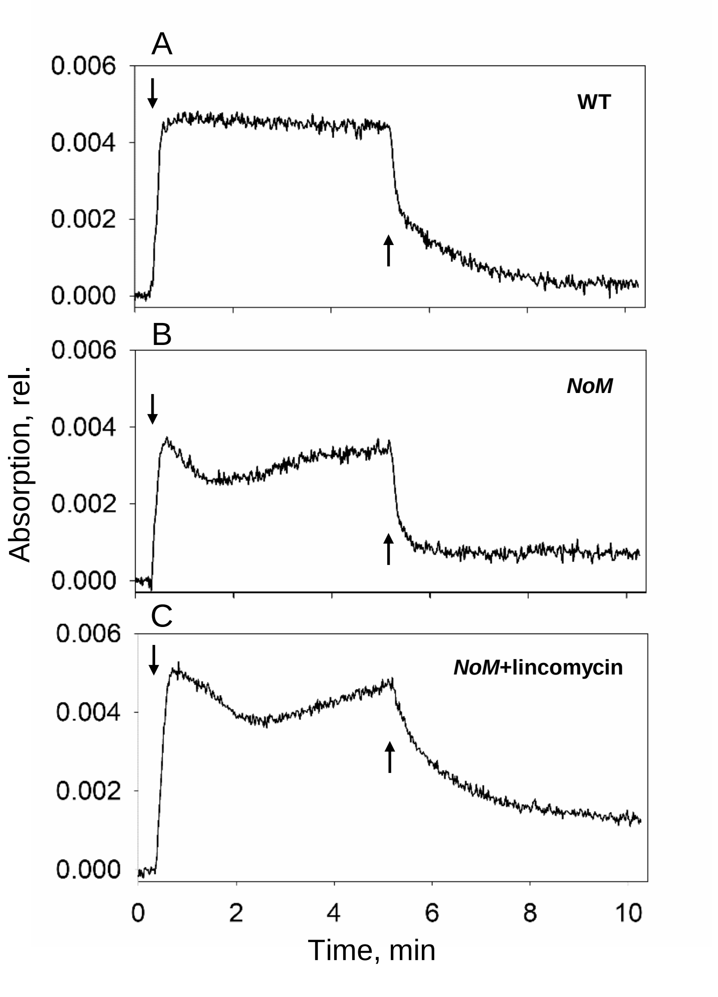

Supplementary Figure 2: Absorption changes related to qE formation, measured in pre-illuminated leaves at 540 nm. Downward arrow indicates actinic light on (900 µmol m^{-2} s^{-1}); upward arrow indicates actinic light off. Traces are averages of 4 independent measurements.
